# miR-9 and miR-124 synergistically affect regulation of dendritic branching via the AKT/GSK3β pathway by targeting Rap2a

**DOI:** 10.1038/srep26781

**Published:** 2016-05-25

**Authors:** Qian Xue, Caiyong Yu, Yan Wang, Ling Liu, Kun Zhang, Chao Fang, Fangfang Liu, Ganlan Bian, Bing Song, Angang Yang, Gong Ju, Jian Wang

**Affiliations:** 1 Institute of Neurosciences, the Fourth Military Medical University, Xi’an 710032, China; 2 Oral and maxillofacial surgery, Stomatology Hospital of Xi’an Jiaotong University, 710004, China; 3 Cardiff Institute of Tissue Engineering & Repair, School of Dentistry, Cardiff University, Cardiff, CF14 4XY, UK; 4 Department of Immunology, the Fourth Military Medical University, Xi’an 710032, China

## Abstract

A single microRNA (miRNA) can regulate expression of multiple proteins, and expression of an individual protein may be controlled by numerous miRNAs. This regulatory pattern strongly suggests that synergistic effects of miRNAs play critical roles in regulating biological processes. miR-9 and miR-124, two of the most abundant miRNAs in the mammalian nervous system, have important functions in neuronal development. In this study, we identified the small GTP-binding protein Rap2a as a common target of both miR-9 and miR-124. miR-9 and miR-124 together, but neither miRNA alone, strongly suppressed Rap2a, thereby promoting neuronal differentiation of neural stem cells (NSCs) and dendritic branching of differentiated neurons. Rap2a also diminished the dendritic complexity of mature neurons by decreasing the levels of pAKT and pGSK3β. Our results reveal a novel pathway in which miR-9 and miR-124 synergistically repress expression of Rap2a to sustain homeostatic dendritic complexity during neuronal development and maturation.

The Ras superfamily consists of highly conserved small GTP-binding proteins that function as genetic switches to control cell proliferation, differentiation, adhesion, and survival. Some members of the Ras superfamily are key regulators of neuronal development and synaptic plasticity^1^,^2^,^3^. The Rap GTP-binding proteins, a subfamily of the Ras superfamily, mediate various biological functions in the hematopoietic, immune, and nervous systems^4^,^5^. The Rap family has five members: Rap1a, Rap1b, Rap2a, Rap2b, and Rap2c^4^. In the nervous system, the Rap proteins are involved in neuronal polarity, synaptogenesis, and synaptic plasticity. In particular, Rap1b plays important roles in establishment of neuronal polarity^6^,^7^,^8^,^9^,^10^, and Rap2a causes spine loss and dendritic shortening^11^.

As posttranscriptional regulators of gene expression expressed in all tissues, miRNAs are involved in control of almost all physiological and pathologic processes, including differentiation, proliferation, apoptosis, development, inflammation, and cancer. MiRNAs also play important roles in the central nervous system, where they are involved in neuronal development and biological functions. MiR-134 controls spine development by targeting the mRNA encoding the protein kinase Limk1, thereby regulating memory and plasticity^12^. MiR-132 promotes dendritic morphogenesis in hippocampal neurons and controls the circadian clock in mice^13^,^14^,^15^. MiR-138, which is enriched in the brain, negatively regulates the size of dendritic spines^16^.

MiR-9 and miR-124, two highly conserved miRNAs that are most abundantly expressed in the mammalian nervous system, both play critical roles in controlling neuron fate and synaptic morphology. miR-9 negatively regulates proliferation of neural stem cells (NSCs) and promotes their neuronal differentiation^17^,^18^. MiR-9 controls axonal extension and branching by regulating Map1b in neurogenesis^19^. MiR-124 is upregulated during neuronal differentiation, suggesting that it plays an important role in this process. MiR-124 represses translation of a large number of non-neuronal transcripts, indicating that it plays a role in maintaining neuronal characteristics^20^. Knockdown of miR-124 results in a ~30% decrease in the total number of postmitotic neurons and an increase in the total number of dividing cells^21^. Furthermore, miR-124 and miR-9 regulate neural lineage differentiation in embryonic stem cells *in vitro*^22^.

Synergism between miR-9/9^*^ and miR-124 mediates the conversion of human fibroblasts to neurons, but separate expression of these miRNAs has no effect^23^,^24^,^25^. MiR-9* and miR-124 reduce proliferation of neural progenitors by repressing the Brg/Brm-associated factor BAF53a, which in turn represses its neuron-specific homolog BAF53b^26^,^27^, a critical factor in dendritic development. Although miR-9 and miR-124 have some distinct targets, their synergistic effects on neuronal development are still not clear and merit further investigation. In this study, we identified Rap2a as a common target gene of miR-9 and miR-124. Moreover, we found that repression of Rap2a by miR-9 and miR-124 affects the activation of AKT and GSK3β, which control neuronal differentiation and dendritic branching. Our findings reveal a novel pathway that governs dendritic branching via the synergistic effects of miR-9 and miR-124.

## Results

### MiR-9 and miR-124 synergistically promote dendritic branching of differentiated neurons, and Rap2a is predicted to be a common target of both miRNAs

**Figure 1:**
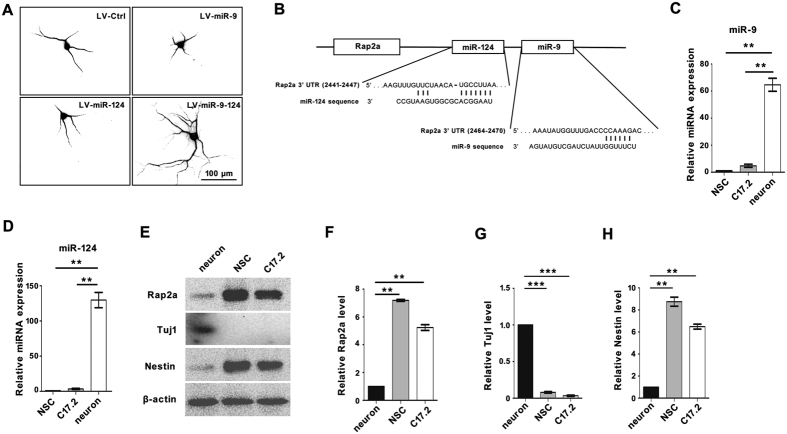
Experimental suggestion of Rap2a as a common target of miR9 and miR-124. (**A**) Dendritic morphology of neurons differentiated from NSCs transfected with LV-Ctrl, LV-miR-9, LV-miR-124, or LV-miR-9-124 for 7 days. Scale bar, 100 μm. (**B**) Schematic representation of the putative base-pairing interactions of miR-9 and miR-124 with the 3′ UTR of Rap2a. qPCR analysis of miR-9 (**C**) and miR-124 (**D**) expression in NSCs, C17.2 cells, and mature neurons. Western blot analysis (**E**) and quantitation by densitometry (**F**) for Rap2a, Tuj1 (**J**), and nestin (**H**) in mature neurons, NSCs, and C17.2 cells; signals were normalized to β-actin. (**P < 0.01; ***P < 0.001).

Previous studies demonstrated that miR-9 and miR-124 play crucial roles in determining neuron fate. In addition, both of these miRNAs start to be expressed at almost the same time, and their levels gradually increase over the course of neuronal development^22^,^28^,^29^. These observations suggest that miR-9 and miR-124 have synergistic effects on neural development. Therefore, we transfected NSCs *in vitro* with lentiviruses that overexpress miR-9, miR-124, or both (Fig. 1A and Supplementary Fig. S1B). Surprisingly, MAP2-positive neurons derived from NSCs co-overexpressing of miR-9 and miR-124 for 7 days had many more dendritic branches than those transfected with control virus or virus expressing miR-9 or miR-124 alone (Fig. 1A). These results suggest that miR-9 and miR-124 can synergistically regulate neurites morphology and promote dendritic branching.

To screen for target genes of miR-9 and miR-124, we used the online prediction tools TargetScan and PicTar^30^,^31^,^32^. Several Ras superfamily members were predicted to be the targets of miR-9 or miR-124 (Table 1). Among them, Rhog was previously verified as a target of miR-124 and shown to control axonal and dendritic branching^33^,^34^. This observation suggested that miR-9 and miR-124 regulate dendritic branching through the Ras superfamily members. Both algorithms strongly predicted that Rap2a is a common target of miR-9 and miR-124 (Table 1). Sequence analysis revealed that the 3′ UTR of Rap2a contains regions complementary to the seed regions of miR-9 and miR-124 (Fig. 1B), i.e., that the Rap2a mRNA has putative miR-9 and miR-124 binding sites in its 3′ UTR (Fig. 1B).

To determine the expression patterns of miR-9, miR-124, and Rap2a, we measured the levels of miR-9 and miR-124 in NSCs, the undifferentiated multipotent neural progenitor cell line C17.2, and mature neurons. The levels of miR-9 and miR-124 were considerable higher in postmitotic neurons than in NSCs or C17.2 cells (Fig. 1C,D). On the contrary, the level of Rap2a was much lower in postmitotic neurons than in NSC and C17.2 cells (Fig. 1E,F). Mature neurons contained a higher level of Tuj1 and lower level of nestin than NSC and C17.2 cells (Fig. 1G,H). The inverted expression patterns of miR-9/-124 and Rap2a supported our hypothesis that Rap2a is a common target of both of these miRNAs.

### Confirmation of Rap2a as a common target of miR-9 and miR-124

To determine whether miR-9 and miR-124 directly repress the Rap2a protein level, we constructed pCAG-miRNA expression plasmids (pCAG-miREPs) pCAG-miR-9, pCAG-miR-124, and pCAG-miR-9-124, in which pri-miR-9, pri-miR-124, or both the pri-miR-9 and pri-miR-124 sequences were placed under the control of the CAG promoter (Supplementary Fig. S1A). All of these plasmids efficiently expressed high levels of the corresponding miRNAs (data not shown).

**Figure 2:**
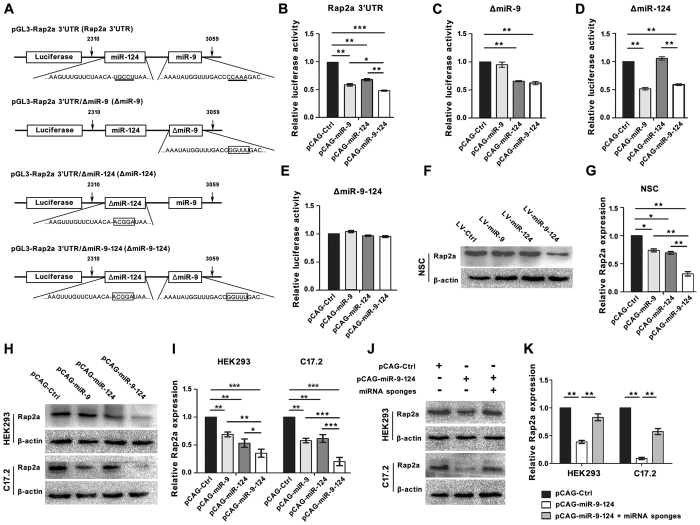
Confirmation of Rap2a as the common target of miR-9 and miR-124. (**A**) Schematic representation of the four reporter plasmids. pGL3-Rap2a 3′UTR (Rap2a 3′UTR): Rap2a 3′ UTR (2310-3059 bp) containing miR-9 and miR-124 binding sites was cloned downstream of luciferase. Underlined bases are sequences complementary to the seed regions of miR-9 and miR-124. pGL3-Rap2a 3′UTR/miR-9 (ΔmiR-9): pGL-Rap2a 3′UTR with a mutation in the miR-9 binding site. pGL3-Rap2a 3′UTR/miR-124 (ΔmiR-124): pGL-Rap2a 3′UTR with a mutation in the miR-124 binding site. pGL3-Rap2a 3′UTR/miR-9-124 (ΔmiR-9-124): pGL-Rap2a 3′UTR with mutations in both the miR-9 and miR-124 binding sites. The boxed bases indicate mutations in sequences complementary to the seed regions of miR-9 and miR-124. (**B–E**) Luciferase activity in HEK293 cells co-transfected with Rap2a 3′ UTR (**B**), ΔmiR-9 (**C**), ΔmiR-124 (**D**), or ΔmiR-9-124 (**E**) reporter plasmid with four miR-EPs. *Firefly* luciferase data were normalized to *renilla* luciferase data. (**F,G**) Western blot analysis (**F**) and quantitation by densitometry (**G**) for Rap2a in NSCs transfected with four miR-EPs. (**H,I**) Western blot analysis (**H**) and quantitation by densitometry (**I**) for Rap2a in HEK293 and C17.2 cells transfected with four miR-EPs. (**J,K**) Western blot analysis (**J**) and quantitation by densitometry (**K**) for Rap2a in HEK293 and C17.2 cells transfected with miR-9-124 and miRNA sponge. Signals were normalized to β-actin. (*P < 0.05; **P < 0.01; ***P < 0.001).

We also constructed four reporter plasmids containing the luciferase cDNA sequence fused to the Rap2a 3′UTR with intact miR-9 and miR-124 binding sites (Rap2a 3′UTR), a mutated miR-9 binding site (named as ΔmiR-9), a mutated miR-124 binding site (named as ΔmiR-124), or mutations in both the miR-9 and miR-124 binding sites (ΔmiR-9-124) (Fig. 2A). After co-transfection of individual reporter plasmids containing the pCAG-miREPs into HEK293 cells harboring the Rap2a 3′UTR reporter, we found that either pCAG-miR-9 or pCAG-124 efficiently suppressed the activity of luciferase relative to pCAG-Ctrl (Fig. 2B). Moreover, pCAG-miR-9-124 suppressed luciferase activity to a greater extent than pCAG-miR-9 or pCAG-124 plasmid (Fig. 2B). However, neither pCAG-miR-9 nor pCAG-miR-124 suppressed luciferase activity in cells carrying a reporter in which its binding site was mutated (i.e., ΔmiR-9 and ΔmiR-124, respectively) (Fig. 2C,D), whereas both suppressed the reporter with the reciprocal mutation in the binding site for the other miRNA (Fig. 2C,D). None of the pCAG-miREPs could suppress the activity of luciferase in ΔmiR-9-124 (Fig. 2E). These results indicate that mutation of the sequences complementary to miRNA seed regions in the Rap2a 3′UTR can efficiently abolish the suppressive activity of miR-9 and miR-124. Moreover, miR-9 and miR-124 synergistically suppressed the Rap2a 3′UTR together, both miRNAs exerted a greater than additive effect on expression.

In addition, we also constructed LV-miREPs in lentivirus: LV-Ctrl, LV-miR-9, LV-miR-124, and LV-miR-9-124 (Supplementary Fig. S1B). LV-miR-9-124 repressed the protein level of Rap2a in NSCs significantly more effectively than either LV-miR-9 (32 ± 4% vs 74 ± 3%, P = 0.0046) or miR-124 (32 ± 4% vs 69 ± 2%, P = 0.0015) (Fig. 2F,G). Since the target sites of miR-9 and miR-124 in Rap2a 3′ UTR sequence were conserved among the species (Supplementary Fig. S2), we transfected pCAG-miREPs into HEK293 and C17.2 cells to further confirm that miR-9 and miR-124 can directly repress Rap2a protein expression. Either pCAG-miR-9 or pCAG-miR-124 repressed the expression of Rap2a in both HEK293 and C17.2 cells (Fig. 2H,I). The Rap2a protein level was more reduced by pCAG-miR-9-124 than by either pCAG-miR-9 or pCAG-miR-124 alone (Fig. 2H,I). The synergistic suppressive effect of miR-9 and miR-124 on Rap2a was abolished by miR-9 and miR-124 sponges (miRNA sponges), which contain eight tandem binding sites for either miR-9 or miR-124, respectively (Fig. 2J,K and Supplementary Fig. S3A–C). Together, we demonstrated that Rap2a is a common target of miR-9 and miR-124, and that miR-9 and miR-124 exert a synergistic effect on the suppression of Rap2a in cells.

### MiR-9 and miR-124 synergistically promote neuronal differentiation and dendritic complexity of NSCs by directly repressing Rap2a

**Figure 3:**
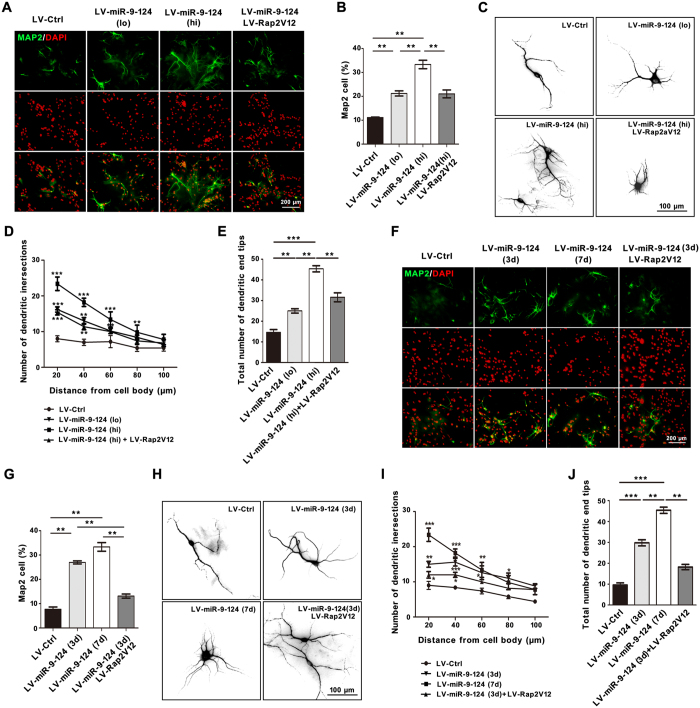
miR-9 and miR-124 synergistically regulate neuronal differentiation and dendritic branching of NSCs by repressing Rap2a. (**A,B**) Representative profiles (**A**) and the percentage (**B**) of MAP2-positive differentiated neurons after transfection of NSCs with miR-9-124 at different viral titers and rescue by Rap2V12. Scale bar, 200 μm. (**C**) Typical dendritic morphology of differentiated neurons after transfection of NSCs with miR-9-124 at different viral titers and rescue by Rap2V12. Scale bar, 100 μm. (**D**,**E**) Sholl analysis of NDIs (**D**) and TNDETs (**E**) of dendritic complexity in differentiated neurons in (**C**) (n = 30 neurons). (**F**,**G**) Representative profiles (**F**) and percentage (**G**) of MAP2-positive differentiated neurons after transfection of NSCs with miR-9-124 for different culture times and rescue by Rap2V12. Scale bar, 200 μm. (**H**) Typical dendritic morphology of differentiated neurons after transfection of NSCs with miR-9-124 for different culture times and rescue by Rap2V12. Scale bar, 100 μm. (**I**,**J**) Sholl analysis of NDIs (**I**) and TNDETs (**J**) of dendritic complexity of differentiated neurons in (**H**) (n = 30 neurons). (*P < 0.05; **P < 0.01; ***P < 0.001).

To examine the synergistic effects of neuronal differentiation and the dendritic complexity of differentiated neurons, we transfected LV-miR-9-124 into NSCs. In this experiment, low and high titers of lentivirus of LV-miR-9-124 were used to infect NSCs (Fig. 3A). After 7 days of culture, LV-miR-9-124 promoted more differentiation of NSCs into MAP2-positive neurons than the control virus (Fig. 3B). More cells were MAP2-positive when a higher viral titer was used (33% ± 3.2% vs 21% ± 2.7%, P = 0.0078) (Fig. 3B). After treatment with LV-Rap2V12 (Supplementary Fig. S1C), a constitutively active form of Rap2a, in combination with LV-miR-9-124, the number of MAP2-positive cells significantly decreased relative to that in cells treated with LV-miR-9-124 alone (33% ± 3.2% vs 20% ± 3%, P = 0.006) (Fig. 3B). We also detected another postmitotic neuron marker NeuN in differentiated neurons after LV-miR-9-12 transfected into NSCs. The numbers of NeuN-positive cells was consistent with MAP2-positive cells in neuronal differentiation (Supplementary Fig. S4).

We also analyzed the dendritic complexity of differentiated neurons following transfection with LV-miR-EPs. The complexity of dendritic branching was analyzed in terms of in morphology, number of dendritic intersections (NDIs), and the total number of dendritic end tips (TNDEPs) (Fig. 3C–E). MAP2-positive neurons derived from NSCs had more dendritic branches, NDIs, and TNDEPs in the LV-miR-9-miR-124 (hi) group than in the LV-Ctrl and LV-miR-9-124 (lo) group (Fig. 3C–E). Rap2V12 decreased the dendritic complexity of neurons transfected with LV-miR-9-124 (hi) (Fig. 3C–E). These findings suggest that miR-9 and miR-124, in a concentration-dependent manner, synergistically regulate the neuronal differentiation of NSCs and dendritic complexity of differentiated neurons. Furthermore, increasing the activity of Rap2a can diminish the synergistic effects of miR-9 and miR-124 on neuronal differentiation and dendritic branching.

Next, we investigated the influence of culture time on the synergistic effects of miR-9 and miR-124 in NSCs. Both 3 and 7 days after transfection with LV-miR-9-124 [miR-9-124 (3d) and miR-9-124 (7d), respectively], NSC cultures contained more MAP2-positive cells than controls (Fig. 3F,G). In addition, dendritic complexity of MAP2-positive cells increased over time following miR-9-124 transfection (Fig. 3H–J). However, LV-Rap2V12 also significantly decreased (P = 0.008) the number of MAP2-positive cells three days after LV-miR-9-124 transfection (Fig. 3F,G). These results suggest that miR-9 and miR-124 synergistically regulate the neuronal differentiation of NSCs and dendritic complexity of differentiated neurons in a time-dependent manner. However, elevated Rap2a activity could also diminish the synergistic effects of miR-9 and miR-124 on the dendritic complexity of MAP2-positive differentiated neurons. Thus, our results demonstrate that miR-9 and miR-124 promote neuronal differentiation of NSCs and increase dendritic branching by inhibiting Rap2a protein.

### Rap2a Reduce dendritic complexity of mature neurons

**Figure 4:**
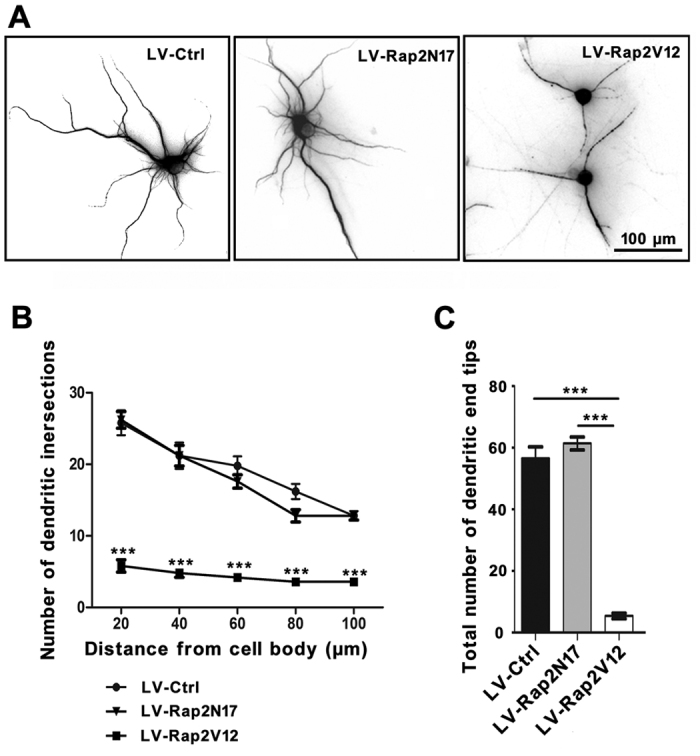
Rap2a repressed dendritic branching in mature neurons. (**A**) Typical dendritic morphology of mature neurons after transfection with LV-Ctrl, Rap2N17 or Rap2V12 for seven days. Scale bar, 100 μm; (**B,C**) Sholl analysis in NDIs (**B**) and TNDETs (**C**) of dendritic complexity in mature neurons in (**A**). (n = 30 neurons, ***P < 0.001).

To further examine the importance of Rap2a inhibited by miR-9 and miR-124 in mature neurons, we transfected postmitotic neurons isolated from cortex with lentivirus expressing LV-Ctrl, LV-Rap2N17 (a dominant-negative mutant of Rap2a protein) or LV-Rap2V12 (Supplementary Fig. S1C), respectively. Seven days after transfected, the postmitotic neurons transfected with LV-Rap2N17 maintained dendritic branch morphology similar to that of LV-Ctrl-transfected neurons (Fig. 4A, left panel and middle panel). Dendritic analysis revealed that neither NDIs nor TNDEPs differed between LC-Ctrl- and LV-Rap2N17-transfected neurons (Fig. 4B,C). In LV-Rap2V12-transfected cells (Fig. 4A, right panel), the number of neuronal dendritic branches was strikingly reduced relative to those in LV-Ctrl- and LV-Rap2N17-transfected cells (Fig. 4A–C). These results suggested that inhibition of Rap2a is indispensable for dendritic branching and complexity of mature neurons.

### AKT-GSK3β signal pathway is involved in the regulation of dendritic complexity of mature neurons by Rap2a

**Figure 5:**
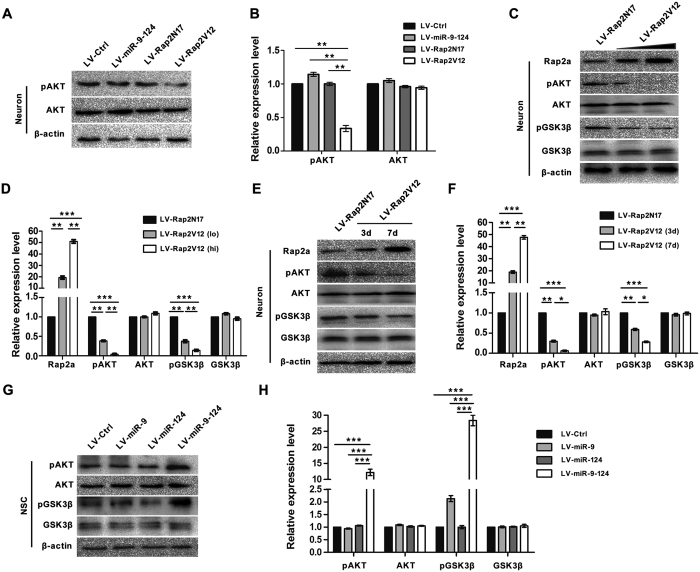
Loss of Rap2a leads to enhanced AKT-GSK3β signaling pathway. (**A,B**) Western blot analysis (**A**) and quantitation by densitometry (**B**) for pAKT (Ser473) and total AKT of mature neurons after transfection with LV-Ctrl, Rap2N17, Rap2V12, or miR-9-124. (**C,D**) Western blot analysis (**C**) and quantitation by densitometry (**D**) for Rap2a, pAKT (Ser473), total AKT, pGSK3β (Ser9) and total GSK3β in mature neurons transfected with Rap2V12 at different viral titers. (E and F) Western blot analysis (**E**) and quantitation by densitometry (**F**) for Rap2a, pAKT (Ser473), total AKT, pGSK3β (Ser9) and total GSK3β in mature neurons after Rap2V12 transfection for different culture times. (**G,H**) Western blot analysis (**G**) and quantitation by densitometry (**H**) for pAKT (Ser473) total AKT, pGSK3β (Ser9) and total GSK3β in NSCs after transfection with LV-Ctrl, miR-9, miR-124, and miR-9-124 transfection. All signals were normalized to β-actin. (*P < 0.05; **P < 0.01; ***P < 0.001).

To identify the signaling pathway(s) involved in the regulation of dendritic complexity by Rap2a, we overexpressed miR-9-124, Rap2N17, and Rap2V12 in neurons. LV-Rap2V12 transfection considerable decreased the level of pAKT in mature neurons relative to LV-Ctrl, LV-miR-9-124, and LV-Rap2N17 transfection (Fig. 5A,B). Thus, Rap2a, but not miR-9 or miR-124, can change the level of pAKT, as mature neurons maintained high levels of miR-9 and miR-124 and a low level of Rap2a (Fig. 1C–F). This result also suggests that the AKT signaling pathway is involved in the regulation of dendritic complexity of mature neurons by Rap2a.

Glycogen synthase kinase 3 beta (GSK3β) acts downstream of Akt, and its activity is inhibited via phosphorylation of its serine 9 residue (Ser9) by pAKT, leading to control of neurogenesis, neuronal polarization, and axonal outgrowth^35^. To further detect the influence of Rap2a on the activity of AKT and GSK3β, we forced mature neurons to overexpress Rap2a. Compared to the LV-Rap2N17 control, overexpression of Rap2V12 resulted in greater reductions in the levels of pAKT and pGSK-3β (Fig. 5C,D). This inhibition pattern was also apparent in LV-Rap2V12-transfected neurons cultivated for longer periods (Fig. 5E,F). Because miR-9 and miR-124 synergistically inhibited Rap2a translation, and NSCs contained low levels of miR-9 and miR-124 and high level of Rap2a (Fig. 1E,F), we wondered whether miR-9 and miR-124 could synergistically alter the levels of pAKT and pGSK-3β in NSCs. Neither miR-9 nor miR-124 could change the levels of pAKT or pGSK-3β in NSCs following transfection with LV-miR-EPs (Fig. 5G,H); only LV-miR-9-124 transfection could significantly increase the levels of pAKT (P = 0.0009) and pGSK-3β (P = 0.0008) in NSCs (Fig. 5G,H). These results further demonstrate that Rap2a, the common target of miR-9 and miR-124, exerts its physical roles in NSCs and neurons by regulating the activity of AKT and GSK3β.

## Discussion

Relationships between miRNAs and targets can be both one-to-many and many-to-one, i.e., one miRNA can repress many proteins, and one protein can be regulated by many miRNAs. For example, miR-155 can target the bone morphogenetic protein (BMP)-responsive transcriptional factors SMAD2 and SMAD5, nuclear factor κB (NF-κB) inhibitor κB-Ras1, and MyD88 to modulate macrophage responses, lymphomagenesis, hematopoiesis, and inflammation^36^,^37^,^38^,^39^. On the other hand, miR-15 and miR-16 control apoptosis by targeting BCL-2 mRNA^40^. MiR-224 and miR-203 downregulate NPAS4 (Neuronal Per-ARNT-SIM homology domain 4) expression through its 3′UTR^41^. This characteristic of miRNAs and their targets has drawn increasing attention to the synergistic effects of miRNAs. For instance, miR-499 and miR-133 synergistically promote cardiac differentiation^42^. Likewise, the combined action of miR-106b, miR-93, and miR-25 effectively repress expression of PTEN transcripts in prostate cancer^43^.

In this study, we observed that co-overexpression of miR-9 and miR-124 in NSCs promoted neuronal differentiation and dendritic branching, whereas neither miRNA had an effect, strongly suggesting that miR-9 and miR-124 exert synergistic effects on neuronal differentiation and dendritic tree complexity. Recent studies report that genetic switches responsible for control of neuronal gene expression are targets of both miR-9 and miR-124. MiR-9 targets repressor-element-1-silencing transcription factor (REST), and miR-9* targets CoREST^44^. MiR-124 also targets CoREST to regulate intrinsic temporal changes in RGC growth cone sensitivity and radial migration of projection neurons^45^,^46^. Although these studies proposed that miR-9 and miR-124 play crucial roles in neuron fate, they did not clearly elucidate the synergistic effects. Here, we showed that miR-9 and miR-124 play synergistic roles in neuron fate, and that Rap2a is their common target.

Previous work shows that Rap2a controls dendritic spine morphology and synaptic plasticity^47^,^48^,^49^, and our results were consistent with those observations. We confirmed that Rap2a represses dendritic branching and neuronal differentiation, and found that miR-9 and miR-124 promote neuronal differentiation and dendritic tree complexity by inhibiting Rap2a. In fact, some Ras superfamily members interact with miR-9 and miR-124. For example, miR-9 is suppressed by the Ras/PI3K/AKT axis, resulting in glioblastoma tumorigenicity^50^. Overexpression of miR-124 in differentiating mouse P19 cells promotes neurite outgrowth by regulating the members of Rho GTPase^51^. MiR-124 controls axonal and dendritic development by targeting the small GTPase RhoG. Our results showed that another member of the Ras superfamily is regulated by miR-9 and miR-124. In addition, overexpression of Rap2V12 could not completely offset the synergistic effects of miR-9 and miR-124, leading us to speculate that miR-9 and miR-124 may regulate neuron fate via another mechanism.

The multifunctional serine/threonine kinase GSK3β plays a variety of roles in activity-dependent regulation of dendritic development and maintenance^52^,^53^. Phosphorylation of GSK3β on Tyr216 leads to activation, whereas phosphorylation of Ser9 by AKT results in inactivation^35^,^54^. We found that levels of pAKT (phosphorylation of Ser473) and pGSK3β (phosphorylation of Ser9) were dramatically downregulated by overexpression of Rap2a in mature neurons (Fig. 5A,B). Thus, the AKT/GSK3β signaling pathway is regulated by Rap2a, and miR-9 and miR-124 can control AKT/GSK3β signaling pathway by targeting Rap2a. It is reported that in B cells Rap2V12 reduces Akt activity via PI3K inhibition^55^. Our results proved that Rap2V12 can also repress Akt activity to inhibit neuronal differentiation and dendritic branching in nervous system. Although Rap2a is involved in the JNK and ERK signaling pathways^56^,^57^, we did not detect obvious changes in the levels of pERK or pJNK upon overexpression of miR-9 and miR-124 in NSCs (data not shown). As homologous proteins of Rap2a, Rap2b was reported to closely correlate with cancer^58^. The biological function of Rap2c was still unclear. The roles of both Rap2b and Rap2c have not yet been reported in nervous system. Considering the vital function of Ras superfamily in nervous system, Rap2b and Rap2c may have some novel roles in differentiation of NSCs, which still need to investigate further.

**Figure 6:**
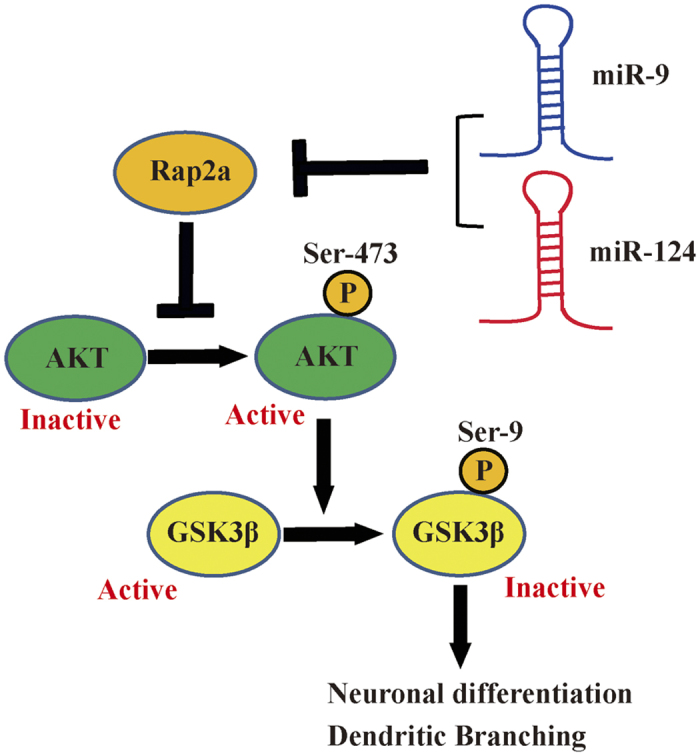
Schematic of miR-9/-124–mediated regulation of neuronal differentiation and dendritic branching by inhibition of Rap2a.

Our results reveal the mechanism by which miR-9 and miR-124 synergistically promote neuronal differentiation and dendritic branching (Fig. 6). Rap2a decreases phosphorylation levels of AKT, thereby inactivating it. MiR-9 and miR-124 repress Rap2a by binding to specific sites in the Rap2a 3′ UTR, thereby releasing the inhibition of AKT, ultimately resulting in inactivation of GSK3β by phosphorylation on Ser9. Inactivation of GSK3β boosts neuronal differentiation and dendritic branching. In short, the results suggest that the synergistic effects of miR-9 and miR-124 control AKT/GSK3β signaling to regulate neuronal differentiation and dendritic complexity by inhibiting Rap2a.

The results of this study reveal a previously unknown interaction between miR-9, miR-124 and Rap2a, and emphasize the synergistic effects of miR-9 and miR-124 on neuronal differentiation and dendritic complexity.

## Materials and Methods

### DNA Constructs and lentivirus preparation

Expression vectors for miR-9 and miR-124 were constructed as described previously^59^. Briefly, the two primary miRNA transcripts (pri-miR-9 and pri-miR-124; specifically, ~500 base pairs around *mmu-miR-9-3* and *mmu-miR-124-1*) were amplified, and either or both of them were cloned downstream of the CAG promoter of pCAG to yield pCAG-miR-9, pCAG-miR-124, and pCAG-miR-9-124 (Supplementary Fig. 1A), or downstream of the EF1 promoter of pCDH-EF1-MCS (System Biosciences, San Diego, CA USA) to yield LV-miR-9, LV-miR-124, and LV-miR-9-124 (Supplementary Fig. 1B).

Vectors for luciferase reporter experiments were established as reported^60^. Bases 2310-3059 of the Rap2a 3′ UTR were amplified by RT-PCR from mouse brain mRNA and inserted downstream of the stop codon of luciferase in vector pGL3 (Promega, Madison, WI, USA). The binding sites in the Rap2a 3′ UTR for miR-9, miR-124, or both (i.e., sequences complementary to bases 2–6 in the miRNA seed regions) were mutated, and the resultant mutant UTRs were inserted downstream of the stop codon of luciferase in pGL3 to yield pGL3-Rap2a, pGL3-Rap2aΔmiR-9, pGL3-Rap2aΔmiR-124, and pGL3-Rap2aΔmiR-9-124 (Fig. 2A).

The *Rap2a* cDNA was amplified from mouse brain using the primer pair 5′-ATGCGCGAGTACAAAGTGG-3′ and 5′-CTATTGTATGTTACAGGCAGAA-3′. To generate dominant-negative Rap2a (Rap2N17) or constitutively active Rap2 (Rap2V12)^57^, a mutant containing a Ser-to-Asn substitution at position 17 (Rap2N17) or Gly-to-Val substitution at position 12 (Rap2V12) was cloned downstream of the EF1 promoter in vector pCDH-EF1-MCS to yield LV-Rap2N17 and LV-Rap2V12 (Supplementary Fig. 1C).

For the miRNA sponge expression vector, eight tandem miR-124 binding sites (Sangon Biotech, Shanghai, China) were ligated into pGL3 (Promega). Likewise, eight tandem mouse miR-9 binding sites were amplified from pBabe-puro-miR-9 sponge (Addgene) and ligated into pGL3 (Supplementary Fig. 2A).

### Cell culture

Human embryonic kidney HEK293 cells were grown in Dulbecco’s Modified Eagle Medium (DMEM) (Gibco, Karlsruhe, Germany) supplemented with 10% fetal bovine serum (Gibco) and 10 mM L-glutamine (Gibco). The multipotent neural progenitor cell line C17.2 was maintained in DMEM supplemented with 10% fetal bovine serum, 5% horse serum (Gibco), and 10 mM L-glutamine. NSCs and neurons were separately established from cortex of embryonic day (E) 14-E16 C57BL/6 mice. Briefly, cortex was microdissected and stripped of meninges, and then tissues were mechanically dissociated into single-cell suspensions. For NSCs, cells were grown in DMEM/F-12 (Gibco) supplemented with 1  mM L-glutamine, 1% N2 supplement (Gibco), 20 μL/mL B-27 supplement minus vitamin A (Gibco), 100 μg/mL penicillin/streptomycin (Gibco), 20 ng/mL epidermal growth factor (EGF), and 20 ng/mL fibroblast growth factor bFGF (PeproTech, London, UK). For neurons, cells were seed in poly-L-lysine-coated plates and grown in serum-free Neurobasal medium (Gibco) supplemented with 10 mM L-glutamine, 100 μg/mL penicillin/streptomycin, and 20 μL/mL B-27 supplement. Cells were maintained in a humidified incubator with 5% CO_2_ at 37 °C.

### RNA extraction and quantitative real-time PCR

For quantitative real-time PCR of miRNA, RNA was extracted with TRIzol (Invitrogen, Carlsbad, CA, USA) and reverse-transcribed with miRNA-specific primers using the miScript Reverse Transcription Kit (Qiagen, Hilden, Germany). Quantitative RT-PCR of mature miRNA was performed using a miRNA-specific primer on a CFX96 Real-Time PCR Detection System (Bio-Rad Laboratories, Hercules, CA, USA). U6 was amplified as a normalization control. Quantitative RT-PCR of miRNAs was performed using the following primers: miR-9, 5′-GGTCTTTGGTTATCTAGCTGTATGA-3′; miR-124, 5′-TTTCCTATGCATATACTTCTTT-3′.

### Luciferase assay

HEK293 cells were seeded in 24-well plates and transfected the next day with 0.4 μg of miRNA expression vector, 0.4 μg of firefly luciferase reporter vector, and 0.08 μg of the control vector pRL-TK (Promega, Madison, USA), which contains *Renilla* luciferase. Transfections were performed using Lipofectamine 2000 (Invitrogen). Each treatment was performed in triplicate in three independent experiments, and the activities of firefly and *Renilla* luciferase were measured consecutively using dual-luciferase assays (Promega) 24 h after transfection.

### Cell transfection and transduction

HEK293 cells and C17.2 cells were seeded in 24-well plates and transfected the next day with miRNA expression vectors with or without miRNA sponges, Transfections were performed using Lipofectamine 2000. The cells were then incubated for 48 h.

For virus transduction, NSCs were digested into single-cell suspensions, and then seeded in poly-L-lysine-coated 24-well plates at 1 × 10^5^cells/cm^2^. The next day, low (5 μL, titer: 1 × 10^8^ TU/mL) or high amounts (10 μL, titer: 1 × 10^8^ TU/mL) of viral supernatants were added to the cells. The medium containing virus was removed and discarded 24 h after transduction and replaced with fresh growth medium of NSCs. Neurons derived from cortex of E14-E16 C57BL/6 mice were plates at 1 × 10^5^cells/cm^2^ and cultured for 3 days. On the fourth day, low (5 μL, titer: 1 × 10^8^ TU/mL) or high amounts (10 μL, titer: 1 × 10^8^ TU/mL) of viral supernatant were added to the cells. The medium containing virus was removed and discarded 24 h after transduction and replaced with fresh growth medium of neurons. The cells were incubated for 3 or 7 days, and then harvested or immunostained.

### Immunocytochemistry

Cells were fixed in 4% paraformaldehyde for 30 min, and then blocked for 1 h with 1% bovine serum albumin containing 0.3% Triton X-100. Blocked cells were incubated overnight at 4 °C with Rabbit polyclonal antibody to MAP2 (Millipore) and Rabbit polyclonal antibody to NeuN antibody (Millipore), and then for 2 h at room temperature with the relative secondary antibodies (DyLight 488-conjugated AffiniPure Donkey anti-rabbit IgG, Jackson ImmunoResearch Laboratories, West Rove, PA, USA). Images were acquired using an IX71 inverted microscope (Olympus, Japan).

### Western blotting

Cells were lysed in lysis buffer (pH 8.0; 50 mM Tris-HCl containing 150 mM NaCl, 5 mM ethylenediaminetetraacetic acid, 1 mM dithiothreitol, 0.5% deoxysodium cholate, 0.1% SDS, 20 μg/mL protease inhibitors aprotinin, 1 mM sodium orthovanadate, 1 mM mercaptoethanol, and 5 mM sodium fluoride), incubated on ice for 30 min, and centrifuged. Protein concentrations in supernatants were determined by Bradford analysis.

Proteins were separated on 10% or 15% (for Rap2a) SDS-PAGE gels at a constant 100 mV voltage and transferred to Polyvinylidene Difluoride (PVDF) membranes at 300 mV for 1 h. PVDF membranes were blocked in 5% nonfat milk for 1 h; incubated overnight at 4 °C with primary antibodies against Rap2a (Proteintech, Wuhan, China), nestin (Sigma-Aldrich, St. Louis, MO, USA), Tuj1 (Sigma-Aldrich), p-AKT (Ser473) (Cell Signaling Technology, Boston, MA), p-GSK3β (Ser9) (Cell Signaling Technology), or β-actin (Sigma-Aldrich); and then incubated for 2 h at room temperature with the relative secondary antibodies conjugated with horseradish peroxidase (Abcam). Immunoreactive bands were visualized using an enhanced chemiluminescence kit on a Bio-Rad Image Lab system.

### Statistical analysis

All statistical analyses of experimental data were performed using GraphPad Prism 5.0 (GraphPad) and are presented as group mean ± SEM. All experiments were repeated at least three times. Comparison of the two groups was performed using independent two-tailed Student’s t tests, and P values < 0.05 were considered significant.

## Additional Information

**How to cite this article**: Xue, Q. *et al.* miR-9 and miR-124 synergistically affect regulation of dendritic branching via the AKT/GSK3β pathway by targeting Rap2a. *Sci. Rep.* **6**, 26781; doi: 10.1038/srep26781 (2016).

## Supplementary Material

Supplementary Information

## Figures and Tables

**Table 1:** Members of the Ras superfamily were predicted as conserved targets of miR-9 and miR-124 by the online prediction tools TargetScan and PicTar.

miR-124	miR-9
Rap2a	Rap2a
Rab34, Rab38	Rab43
Rhog	Rhoq
Raph1	RAS p21 protein activator 2
Rreb1	
Ras repressor protein 1	
Ras-GTPase-activating protein SH3-domain binding protein 1	
