# Supplementary material for: miR-9 and miR-124 synergistically affect regulation of dendritic branching via the AKT/GSK3β pathway by targeting Rap2a
Source: Sci Rep. 2016 May 25;6:26781. doi: 10.1038/srep26781 (PMC4879704; doi:10.1038/srep26781)
Supplement: Supplementary Information [file srep26781-s1.pdf]

miR-9 and miR-124 synergistically affect regulation of dendritic branching via the AKT/GSK3β pathway by targeting Rap2a

Qian Xue,[1, +] Caiyong Yu,[1, +] Yan Wang,[2, +] Ling Liu,[1] Kun Zhang,[1] Chao Fang,[1] Fangfang Liu,[1] Ganlan Bian,[1] Bing Song,[3] Angang Yang,[4] Gong Ju[1, *] and Jian Wang[1, *]

[1]Institute of Neurosciences, the Fourth Military Medical University, Xi'an 710032, China

[2]Oral and maxillofacial surgery, Stomatology Hospital of Xi'an Jiaotong University, 710004, China

[3]Cardiff Institute of Tissue Engineering & Repair, School of Dentistry, Cardiff University, Cardiff, CF14 4XY, UK

[4]Department of Immunology, the Fourth Military Medical University, Xi'an 710032, China

* Corresponding Author: Jian Wang, E-mail: jwangfm@fmmu.edu.cn. Or to: Gong Ju, E-mail: jugong@fmmu.edu.cn

[+] These authors contributed equally to this work.

Supplemental Figure Legends

Supplementary Figure S1. Schematics of expression plasmids

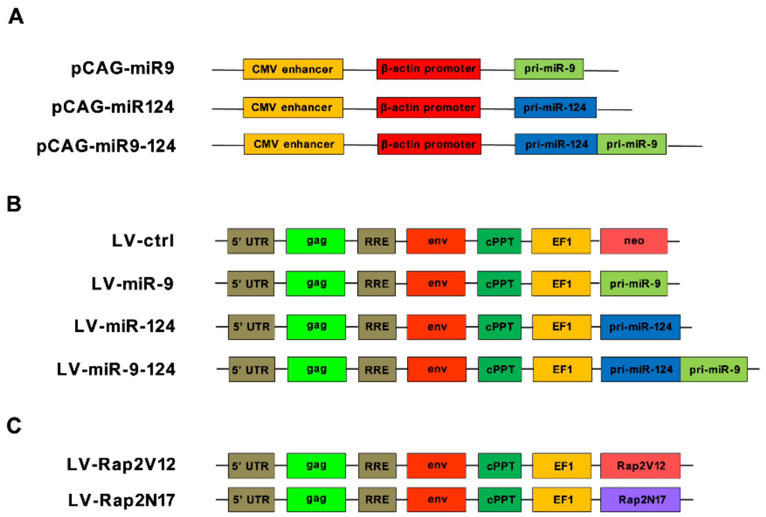

(A) pCAG-miR-EPs were constructed by inserting pri-miR-9, pri-miR-124, or both sequences downstream of the CAG promoter. (B) LV-miR-EPs were constructed by inserting pri-miR-9, pri-miR-124, or both sequences into lentivirus vectors. (C) LV-Rap2V12 and LV-Rap2N17 were constructed by inserting the Rap2V12 and Rap2N17 sequences into lentivirus vectors.

Supplementary Fig S2. The conserved sites in Rap2a 3' UTR sequence targeted by miR-9 and miR-124 among the species were extracted from Targetscan web.

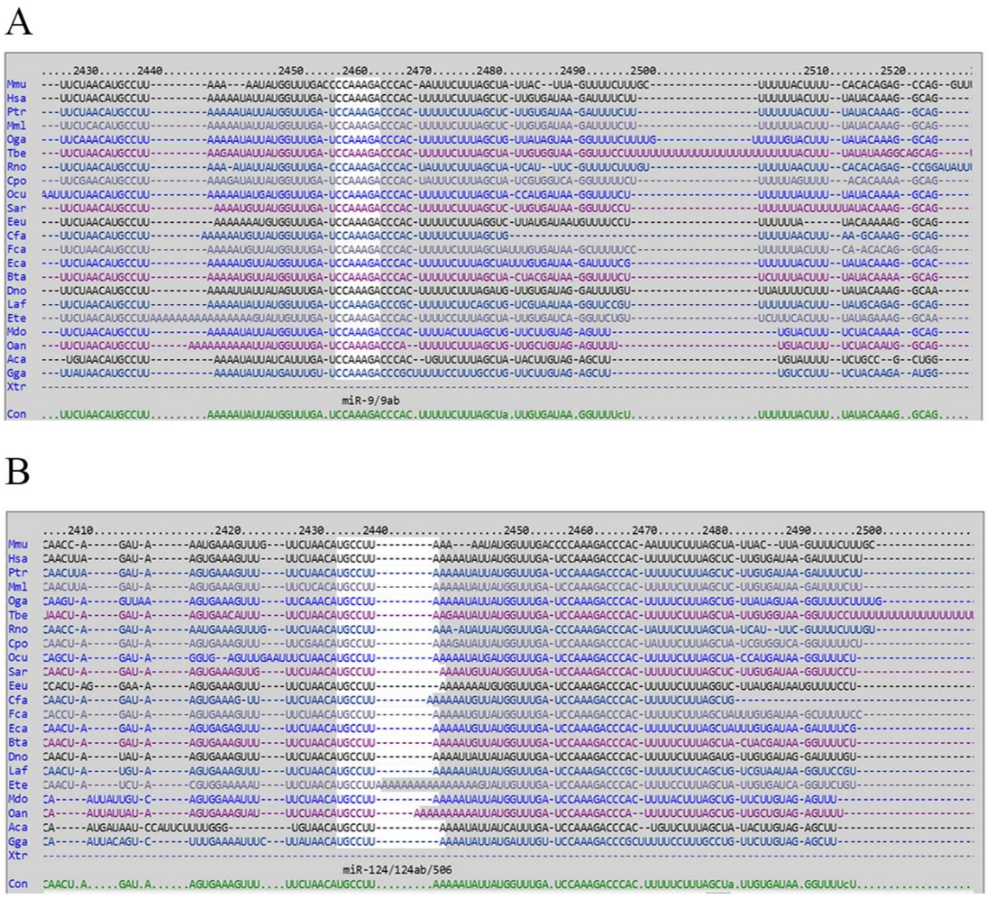

The target sites of miR-9 (A) and miR-124 (B) in Rap2a 3' UTR sequence were conserved among the species. White area are the target sites.

The following is the link to it on Targetscan web[1-2].

http://www.targetscan.org/cgi-bin/targetscan/vert_70/view_gene.cgi?rs=ENST00000245304.4&taxid=9606&showcnc=0&shownc=0&shownc_nc=&showncf=&subset=1

Supplementary Figure S3. Schematics of miRNA sponges.

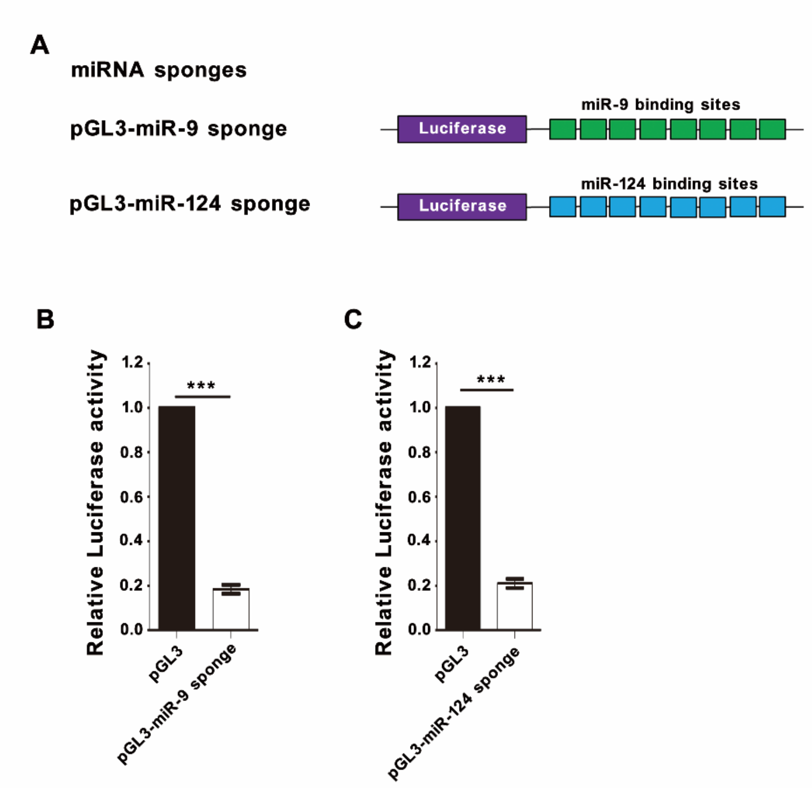

(A) Structures of pGL3-miR-9 and pGL3-miR-124 sponges. (B) Luciferase activity of pGL3-miR-9 sponge was significantly inhibited by miR-9. (C) Luciferase activity of pGL3-miR-124 sponge was significantly inhibited by miR-124. (*** $P < 0.001$).

Supplementary Figure S4. miR-9 and miR-124 synergistically regulate neuronal differentiation

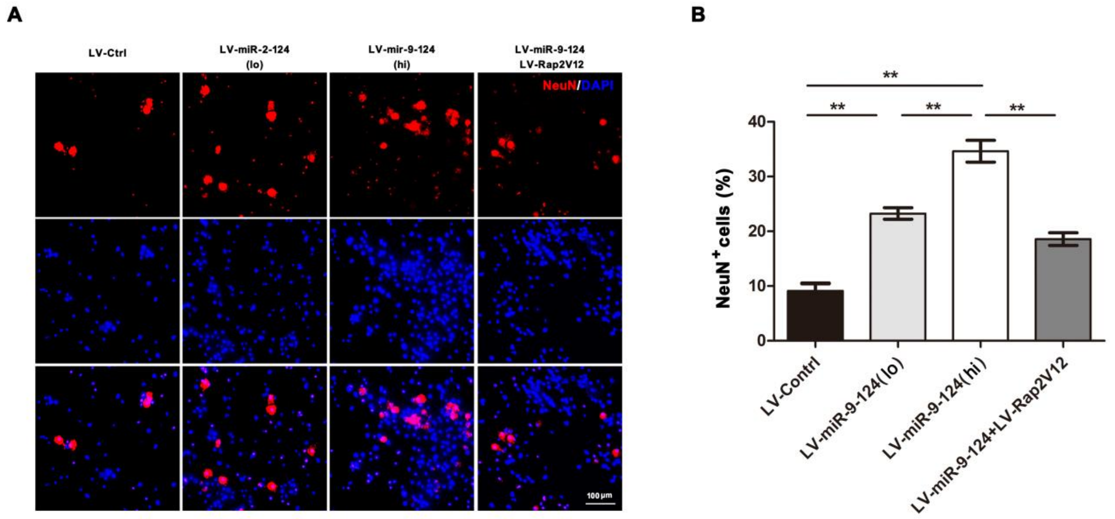

Representative profiles (A) and the percentage (B) of NeuN-positive differentiated neurons after transfection of NSCs with miR-9-124 at different viral titers and rescue by Rap2V12. Scale bar, 100 μm.

References

1. Lewis BP, Burge CB, Bartel DP. Conserved Seed Pairing, Often Flanked by Adenosines, Indicates that Thousands of Human Genes are MicroRNA Targets. Cell 120:15-20 (2005).
2. Friedman RC, Farh KK, Burge CB, Bartel DP. Most Mammalian mRNAs Are Conserved Targets of MicroRNAs. Genome Res. 19:92-105 (2009).
